# The Transmembrane Protein Semi1 Positions Gamete Nuclei for Reciprocal Fertilization in *Tetrahymena*

**DOI:** 10.1016/j.isci.2019.100749

**Published:** 2019-11-28

**Authors:** Takahiko Akematsu, Rosalía Sánchez-Fernández, Felix Kosta, Elisabeth Holzer, Josef Loidl

**Affiliations:** 1 Department of Chromosome Biology, University of Vienna, Dr. Bohr-Gasse 9, Vienna 1030, Austria

**Keywords:** Genetics, Developmental Genetics, Molecular Biology

## Abstract

During sexual reproduction in the ciliate, *Tetrahymena thermophila*, cells of complementary mating type pair (“conjugate”) undergo simultaneous meiosis and fertilize each other. In both mating partners only one of the four meiotic products is “selected” to escape autophagy, and this nucleus divides mitotically to produce two pronuclei. The migrating pronucleus of one cell translocates to the mating partner and fuses with its stationary pronucleus and vice versa. Selection of the designated gametic nucleus was thought to depend on its position within the cell because it always attaches to the junction with the partner cell. Here we show that a transmembrane protein, Semi1, is crucial for attachment. Loss of Semi1 causes failure to attach and consequent infertility. However, a nucleus is selected and gives rise to pronuclei regardless of Semi1 expression, indicating that attachment of a nucleus to the junction is not a precondition for selection but follows the selection process.

## Highlights

• The transmembrane protein Semi1 is specifically expressed in mating *Tetrahymena*
• Semi1 marks the one nucleus out of four meiotic products that gives rise to pronuclei
• Semi1 mediates the attachment of the selected nucleus to the conjugation junction
• Semi1 interacts with Zfr3, which mediates the reciprocal exchange of pronuclei

## Graphical Abstract

**Figure:**
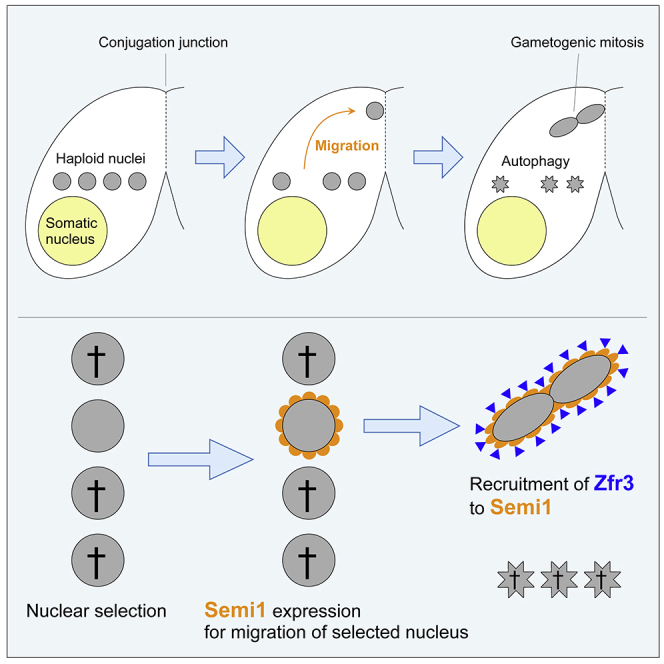

## Introduction

**Figure 1:**
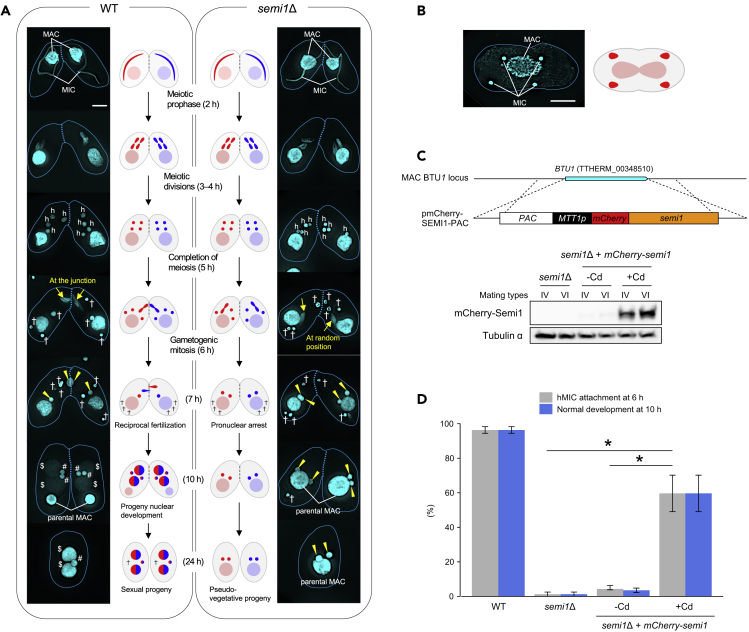
Semi1 Mediates hMIC Attachment to the Conjugation Junction (A) Timeline of conjugation in WT (left) and *semi1*Δ (right) cells (see also Figures S1C and S1D) stained with DAPI, shown as fluorescence microscopy images and schematic diagrams. h: hMICs; arrows: selected hMICs undergoing gametogenic mitosis; †: degenerating unselected hMICs; arrowhead: gametic pronucleus; $: progeny MACs; #: progeny MIC. Dotted line: conjugation junction. (B) Image (left) and diagram (right) of vegetative division of a *semi1*Δ exconjugant, in which unexchanged gametic pronuclei are maintained (see also Figure S1E). Scale bar: 10 μm. (C) Rescue of the *semi1*Δ phenotype by semi1-mCherry expression. The pmCherry-SEMI1-PAC plasmid, containing a puromycin resistance marker (PAC), cadmium-inducible MTT1 promoter, and mCherry-Semi1-expression cassette, was integrated into the MAC *BTU1* locus by homologous recombination. The Western blot shows that mCherry-Semi1 expression is induced by the addition of cadmium. Tubulin ɑ was the loading control. (D) Percentage of cells with normal hMIC attachment at 6 h after the initiation of conjugation (see also Figure S2) and development of progeny nuclei at 10 h. Columns and error bars represent the means and standard deviations of three independent experiments. Asterisk (*) shows a significant difference between means (p < 0.01 as calculated by Tukey's honestly significant difference [HSD] test on RStudio).

The model ciliate *Tetrahymena thermophila* stably maintains different germline and somatic genomes in two separate nuclei within a single cytoplasm. The small diploid micronucleus (MIC), which is essentially transcriptionally silent, contains the germline genome, whereas the large, transcriptionally active polyploid macronucleus (MAC) contains the somatic genome. The phenotype of a cell depends on the genetic constitution of its MAC, whereas only the MIC genome is transmitted to progeny MICs and MACs during sexual reproduction (Figure 1A, left panel), also known as conjugation (Prescott, 1994, Orias et al., 2011). When two cells of complementing sexes (mating types) conjugate, they undergo synchronous meiosis. Meiosis of the MIC produces four identical haploid MICs (hMICs) that are in the G2 phase of the cell cycle due to DNA replication, which takes place concomitantly with meiotic anaphase II (Cole and Sugai, 2012). After meiosis, only one hMIC is selected to form the gamete, whereas the three unselected hMICs are degraded by autophagy (Liu and Yao, 2012). All four meiotic products undergo post-meiotic DNA double-strand break (PM-DSB) formation. DNA damage in hMICs correlates with the appearance of γH2AX foci (Akematsu et al., 2017), which are markers of DSBs (Chowdhury et al., 2005, Kadoch and Crabtree, 2015). The γH2AX foci disappear only from one hMIC, and this occurs at the same time as histone H3 becomes acetylated at lysine 56 (H3K56ac), which is an epigenetic marker of reconstituted chromatin on nascent DNA (Shi and Oberdoerffer, 2012, Chen et al., 2008). Only this hMIC undergoes another round of mitosis, known as gametogenic mitosis, to produce gametic pronuclei (Akematsu et al., 2017). One of the pronuclei migrates to the partner cell to fertilize its stationary pronucleus, whereas the other becomes fertilized by the migratory pronucleus of the partner cell. This reciprocal fertilization leads to the formation of zygotes in both partners. Attenuated PM-DSB formation culminates in autophagy for all hMICs (Akematsu et al., 2017), strongly suggesting that hMIC selection involves self-inflicted DNA damage in all hMICs followed by DNA repair in only one. Indeed, the DNA repair proteins DNAPKcs (involved in DNA repair by non-homologous end-joining) and Rad51 (involved in recombinational repair) and the histone H3-H4 chaperone Asf1 specifically localize to the selected hMIC (Akematsu et al., 2017).

In the related species, *Paramecium caudatum*, the fate of hMICs is proposed to depend on their position in the cell (Yanagi, 1987). In this species, an hMIC that happens to be in contact with the conjugation junction (where the plasma membranes of conjugating cells are fused) may be protected from autophagic degradation by its location in this specific microenvironment and thus able to recruit DNA repair proteins. Indeed in both *P*. *caudatum* (Ishida et al., 1999, Gao et al., 2010) and *T*. *thermophila* (Cole and Sugai, 2012), the hMIC located at the junction is always selected to undergo gametogenic mitosis. However, two fundamental questions remain: (1) how does the hMIC attach to the conjugation junction (hereafter called “hMIC attachment”)? and (2) is hMIC attachment a key molecular switch that controls hMIC selection? Here, we report that the protein Semi1 (selected haploid micronucleus 1) is key to understanding the mechanism of hMIC attachment and the behavior of the selected nucleus.

## Results

### Semi1 Mediates hMIC Attachment to the Conjugation Junction

Semi1 (711 aa, 84 kDa, encoded by TTHERM_00985030; www.ciliate.org) is a putative transmembrane protein in *T*. *thermophila* (Figure S1A) that has no known homolog in other organisms. A genetic screen for genes that are transcriptionally upregulated during the pre-zygotic period of conjugation (Miao et al., 2009) found that *SEMI1* is required for conjugation, and western blotting demonstrated that Semi1 protein is expressed only in conjugating cells (Figure S1B). Somatic knockout (*semi1*Δ) cells of two different sexes (mating types) were produced by co-deletion (co-Del), which uses the natural DNA elimination mechanism of *T*. *thermophila* to target specific sequences with flanking deletion signal motifs (Figures S1C and S1D) (Hayashi and Mochizuki, 2015). DAPI (4′,6-diamidino-2-phenylindole) nuclear staining showed that *semi1*Δ mating cells undergo meiosis and produce four hMICs at 5 h after the initiation of conjugation, similar to wild-type (WT) mating cells (Figure 1A). However, most *semi1*Δ mating cells initiated gametogenic mitosis in an hMIC that was not attached to the conjugation junction at the 6 h time point (Figure 1A). No pronuclei exchange occurred between the mating partners (Figure 1A), and the single hMIC undergoing mitosis in each cell was retained, whereas the unselected hMICs had disappeared by 10 h (Figure 1A). Mating in *semi1*Δ was completed by 24 h, with each progeny cell containing two MICs and the parental MAC (i.e. pseudo-vegetative progeny; Figure 1A). The two MICs were maintained during asexual division of the exconjugants (Figures 1B and S1E). Unlike in the similar process of autogamy in *Paramecium tetraurelia* (Garnier et al., 2004, Komori et al., 2004), self-fertilization did not occur.

Because co-Del can create off-target changes in the genome (Hayashi and Mochizuki, 2015), it was formally possible that the aberrant conjugation phenotype in *semi1*Δ cells could have resulted from off-target mutations. In fact, an analysis showed that about 700 bp extra non-coding sequences were deleted together with the target sequence in both sexes (Figure S1C). To show that deletion of the target gene was responsible for the aberrant conjugation phenotype, an mCherry-tagged Semi1 (mCherry-Semi1) construct expressed under the cadmium-inducible *MTT1* promoter (Shang et al., 2002) was introduced into the non-essential *β-tubulin* genomic locus of *semi1*Δ cells (Figure 1C). Induction of mCherry-Semi1 expression (Figure 1C) partially rescued the phenotype: over 60% of *semi1*Δ + *mCherry-semi1* cells underwent gametogenic mitosis at the conjugation junction and formed progeny nuclei (Figure 1D). Therefore, the aberrant conjugation phenotype in *semi1*Δ is unlikely to result from the off-target effects of co-Del.

**Figure 2:**
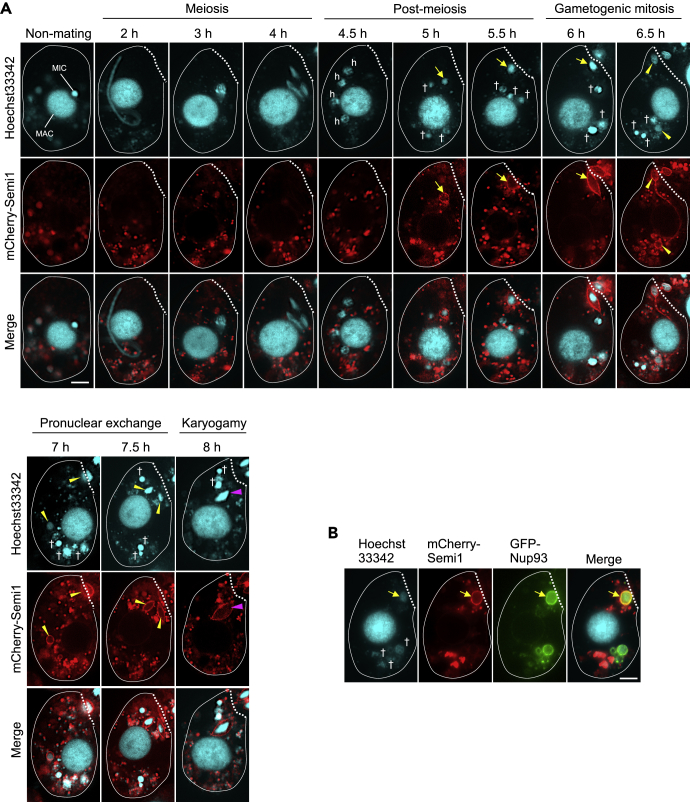
Live Cell Imaging of mCherry-Semi1 Localization (A) mCherry-Semi1 is localized to the hMIC selected for gametogenic mitosis. The bright spheres in the cytoplasm are digestive vacuoles probably incorporating overexpressed or unfolded mCherry-Semi1. (B) mCherry-Semi1 and GFP-Nup3 co-localize at the periphery of the selected hMIC in live cells. h: hMIC; arrow: selected hMIC undergoing gametogenic mitosis; †: degenerating unselected hMIC; yellow arrowheads: gametic pronucleus; magenta arrowhead: fertilized nucleus; dotted line: conjugation junction. Scale bars: 10 μm.

The mCherry-Semi1 construct (Figure 1C) was introduced to WT cells for the localization of Semi1. mCherry-Semi1 localized to a single hMIC (Figure 2A). This was the only nucleus to attach to the conjugation junction, followed by gametogenic mitosis, gametic pronuclear exchange, and karyogamy (Figure 2A), which is characteristic of the selected hMIC. Because mCherry-Semi1 expression in the selected hMIC overlaps with the expression pattern of GFP-tagged Nup93 (GFP-Nup93; Figure 2B) (Iwamoto et al., 2009), Semi1 is likely to be a nuclear membrane protein. Mutagenesis analysis showed that all four hydrophobic regions of Semi1, including the transmembrane helix, were required for its perinuclear localization (Figures S2A–S2E). Moreover, none of the mutant Semi1 proteins could rescue the *semi1*Δ phenotype in co-expression experiments (Figure S2F). We therefore suggest that perinuclear localization of Semi1 is required for regulation of hMIC attachment to the conjugation junction.

### DNA Repair Markers Indicate that hMIC Selection Occurs without hMIC Attachment in *semi1*Δ Cells

**Figure 3:**
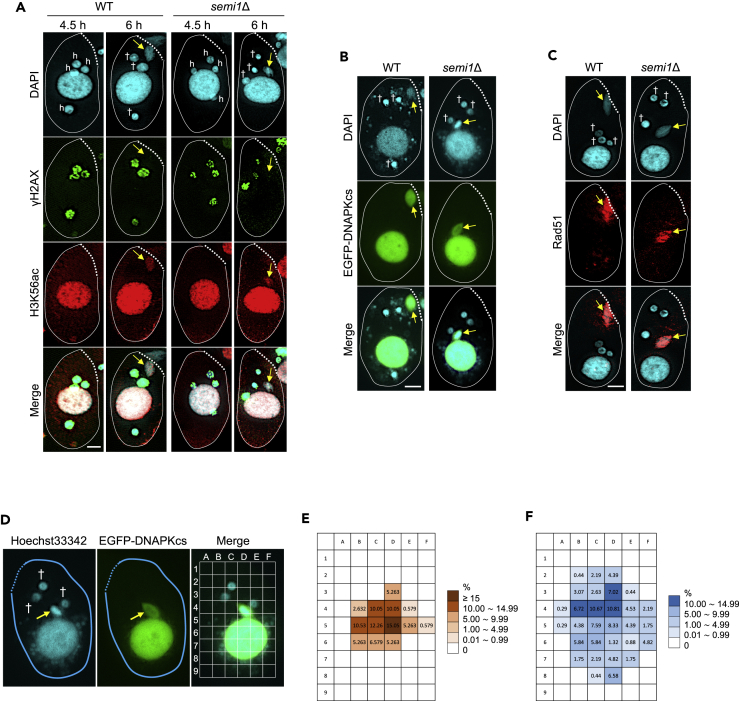
DNA Repair Markers Indicate that hMIC Selection Occurs without hMIC Attachment in *semi1*Δ Cells (A) γH2AX foci are formed in all four hMICs of both WT and *semi1*Δ cells but are lost in only one hMIC (arrow), concomitant with histone H3 acetylation at lysine 56 (H3K56ac). (B) Localization of EGFP-DNAPKcs in a single hMIC (arrow) in both WT and *semi1*Δ cells at 6 h after the initiation of conjugation. (C) Localization of Rad51 in an hMIC (arrow). (D) The position of the selected hMIC (arrow) and unselected hMICs (†) in *semi1*Δ cells expressing EGFP-DNAPKcs was determined using a 9 × 6 grid. (E) Heatmap showing the cytoplasmic distribution of selected hMICs. (F) Heatmap showing the cytoplasmic distribution of unselected hMIC. The heatmaps were based on data from 17 cells with a clearly defined selected MIC and 17 cells with four unselected MICs. h: hMIC; dotted line: conjugation junction; arrow: selected hMICs undergoing gametogenic mitosis; †: degenerating unselected hMIC. Scale bars: 10 μm.

In *P*. *caudatum*, selection of an hMIC is proposed to involve its attachment to the conjugation junction (Yanagi, 1987). If this were also the case in *T*. *thermophila*, then none of the hMICs in *semi1*Δ cells would undergo DNA repair and they all would be degraded. Remarkably, however, one hMIC undergoes mitosis in the *semi1*Δ mutant irrespective of its position within the cell (Figures 1A and 3A). To explore whether this nucleus bears the γH2AX and H3K56ac marks upon repair of PM-DSBs that are characteristic of a selected hMIC (Akematsu et al., 2017), double immunostaining of γH2AX and H3K56ac was performed in *semi1*Δ cells.

We observed γH2AX foci in all four hMICs in *semi1*Δ cells at 4.5 h after the initiation of conjugation, as seen in WT cells (Figure 3A), indicating that PM-DSB formation is independent of Semi1 expression. In contrast, only the MAC displayed H3K56 acetylation, which is consistent with its euchromatic state at this time point (Figure 3A). After 6 h, only one hMIC had initiated gametogenic mitosis in *semi1*Δ cells, and in this hMIC H3K56 acetylation occurred concomitantly with the disappearance of γH2AX (Figure 3A). This result strongly suggests that hMIC attachment to the conjugation junction is not required for DNA repair. Analysis of EGFP-DNAPKcs and Rad51 localization showed that these major DNA repair factors were recruited only to the hMIC undergoing gametogenic mitosis, regardless of Semi1 expression (Figures 3B and 3C).

To test whether positional cues other than association with the conjugation junction may determine the fate of hMICs, we marked the selected hMIC with EGFP-DNAPKcs in *semi1*Δ cells (Figure 3D) and determined its position within the cells. We found that the selected hMIC preferentially resides in the space between the MAC and the conjugation junction (Figure 3E), whereas the unselected hMICs are more evenly distributed throughout the cell (Figure 3F) at the time when they begin to move toward the posterior part, which is highly enriched in lysosomes (Akematsu et al., 2017). Thus, although hMIC selection does not take place in the vicinity of the conjugation junction, its location may not be completely random.

### Semi1 Acts on the MAC in the Absence of hMIC Selection

Given that mCherry-Semi1 localizes exclusively to the selected hMIC (Figure 2A), it is possible that Semi1 may have an affinity for molecules that appear on its nuclear envelope upon hMIC selection. If so, mCherry-Semi1 should not be expressed in the hMIC of mutants in which hMIC selection does not occur. To confirm this, we expressed mCherry-Semi1 in *spo11*Δ cells, where hMIC selection is prevented by PM-DSB suppression (Akematsu et al., 2017).

**Figure 4:**
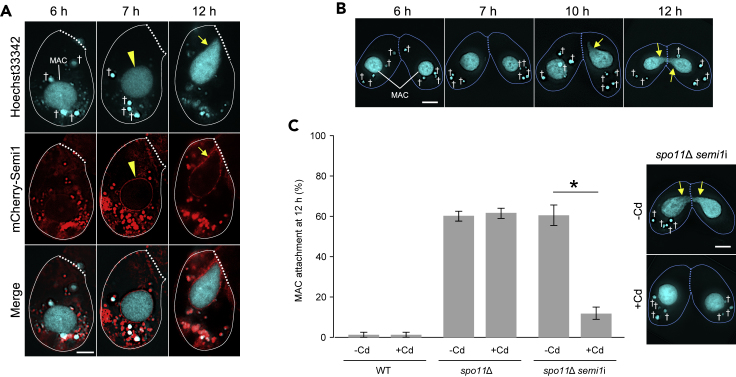
Semi1 Acts on the MAC in the Absence of hMIC Selection (A) Localization of Semi1 in a *spo11*Δ strain, which is defective in hMIC selection. †: degenerating unselected hMIC; arrowhead: MAC bearing mCherry-Semi1; arrows: MAC elongating toward the conjugation junction. (B) In *spo11*Δ cells, MAC attachment to the conjugation junction occurs between 6 h and 12 h after the initiation of conjugation. (C) Left, percentage of cells showing MAC attachment at 12 h after the initiation of conjugation. Columns and error bars represent the means and standard deviations of three independent experiments. Asterisk (*) shows a significant difference (p < 0.01, as calculated by Tukey's HSD test on RStudio). Right, examples of conjugating *spo11*Δ *semi1*i (uninduced) and *spo11*Δ *semi1*i (induced) cells (see also Figure S3). Arrow: selected hMIC undergoing gametogenic mitosis; †: degenerating unselected hMIC; arrowhead: gametic pronucleus; dotted line: conjugation junction. Scale bars: 10 μm.

As predicted, mCherry-Semi1 did not localize to any hMIC in *spo11*Δ cells at 6 h after the initiation of conjugation (Figure 4A), when all hMICs are programmed to degenerate. Notably, a clear mCherry-Semi1 signal became apparent at the periphery of the MAC after 7 h in *spo11*Δ cells (Figure 4A) but not in the WT (Figure 2A). Remarkably, the MAC in the *spo11*Δ cells (i.e. with the mCherry-Semi1 signal) became somewhat elongated and was attached to the conjugation junction at 12 h (Figure 4A). This phenomenon resembles the aberrant MAC elongation toward the conjugation junction seen in the inbred mutant strain B1, in which hMIC selection is defective (Nanney and Nagel, 1964). About 60% of *spo11*Δ cells showed the MAC attachment phenotype (Figures 4B and 4C). To determine whether MAC attachment to the conjugation junction is Semi1 dependent, a cadmium-inducible *semi1* RNA interference (RNAi) construct (*semi1*i; Figure S3) was introduced into *spo11*Δ cells. *semi1*i expression significantly reduced the proportion of cells with MAC attachment (from 60% to 12%; Figure 4C), strongly suggesting that Semi1 attaches the MAC to the conjugation junction instead of the selected hMIC when hMIC selection is lacking.

### Proteomic Analysis of Semi1

Considering its localization and role in attachment to the conjugation junction, Semi1 may mediate the attachment between surface proteins on both the selected hMIC and the conjugation junction. To investigate this possibility, interaction partners of mCherry-Semi1 were co-immunoprecipitated and identified by mass spectrometry (MS). Cells expressing the free mCherry tag (Figure S2A) were used as the control. SAINTexpress analysis (Teo et al., 2014) of the MS data revealed 65 potential interaction partners (p < 0.05) of which 12 appeared to have conjugation-specific expression (Tables 1 and S1). Of these, Zfr3 (Zinc Finger-Related 3; encoded by TTHERM_00531890) was the most abundant (see below). Another constitutively expressed Semi1 interactor was 14-3-3 protein 18 (Ftt18; Table S1). Interestingly, enhanced green fluorescent protein (EGFP)-tagged Ftt18 (Ftt18-EGFP) localized to both the conjugation junction and the periphery of the selected hMIC (Figure S4), suggesting that a strong binding affinity between Semi1 and Ftt18 may generate the cohesive force behind hMIC attachment to the conjugation junction. Indeed, 14-3-3 proteins are known to bind a multitude of functionally diverse proteins, including transmembrane proteins (Fu et al., 2000). Unfortunately, the function of Ftt18 is unknown and was difficult to determine because the *FTT18* gene seems to be essential for vegetative growth. In addition, a specific RNAi construct for *FTT18* was difficult to design because of high sequence similarity between *FTT18* and the other two *FTT* paralogs (TTHERM_00592720 and TTHERM_00160770).

**Table 1:** MS Identification of Conjugation-specific Interaction Partners with Semi1

Gene ID (TTHERM_)	AvgCount	Control Count	P Value	Protein Name	Description
00985030	383	2|33	0	Semi1	Transmembrane protein putative
00531890	45	0|9	0.01	Zfr3	Zinc finger domain containing protein
000158019	22.5	0|1	0	None	Hypothetical protein
00083300	17	0|1	0	None	Cullin family protein
00442210	16	0|6	0.02	Rpn2	RPN1 26S proteasome regulatory complex subunit RPN2
00703970	13.5	0|0	0	Ima5	IMA5 import in subunit alpha putative
00437600	10	0|1	0	None	Succinyl-CoA ligase [GDP-forming] subunit beta
01079260	9.5	0|0	0	None	ATP-dependent metalloprotease FtsH
00221140	9	0|0	0	Ars2	ARS2 alanine-tRNA ligase/alanyl-tRNA synthetase protein
00624630	9	0|3	0.01	None	Transmembrane protein putative
00158020	6	1|1	0.02	None	PCI-domain protein
00444470	5.5	0|1	0	None	S-adenosylmethionine synthase protein
00294640	5	2|1	0.05	None	NADH-ubiquinone oxidoreductase 1 chain putative

See also Table S1 and Figure S4.

### Zfr3-mediated Gametic Pronuclear Exchange Is Dependent on Semi1

The gene encoding Zfr3 is also called *Coi9* (*conjugation-induced gene 9*) because of its conjugation-specific expression (Woehrer et al., 2015). The Zfr3 protein contains a zinc finger structure (Figure S5A) but has no clear homologs in other organisms. A previous knockout study reported that macronuclear *ZFR3* is required for proper conjugation (Xu et al., 2012, Woehrer et al., 2015). However, the timing and mechanism of its role in conjugation was unclear. To further investigate the function of Zfr3 and its relation to Semi1, we drastically reduced Zfr3 expression using *zfr3* RNAi (*zfr3*i; Figure S5B).

**Figure 5:**
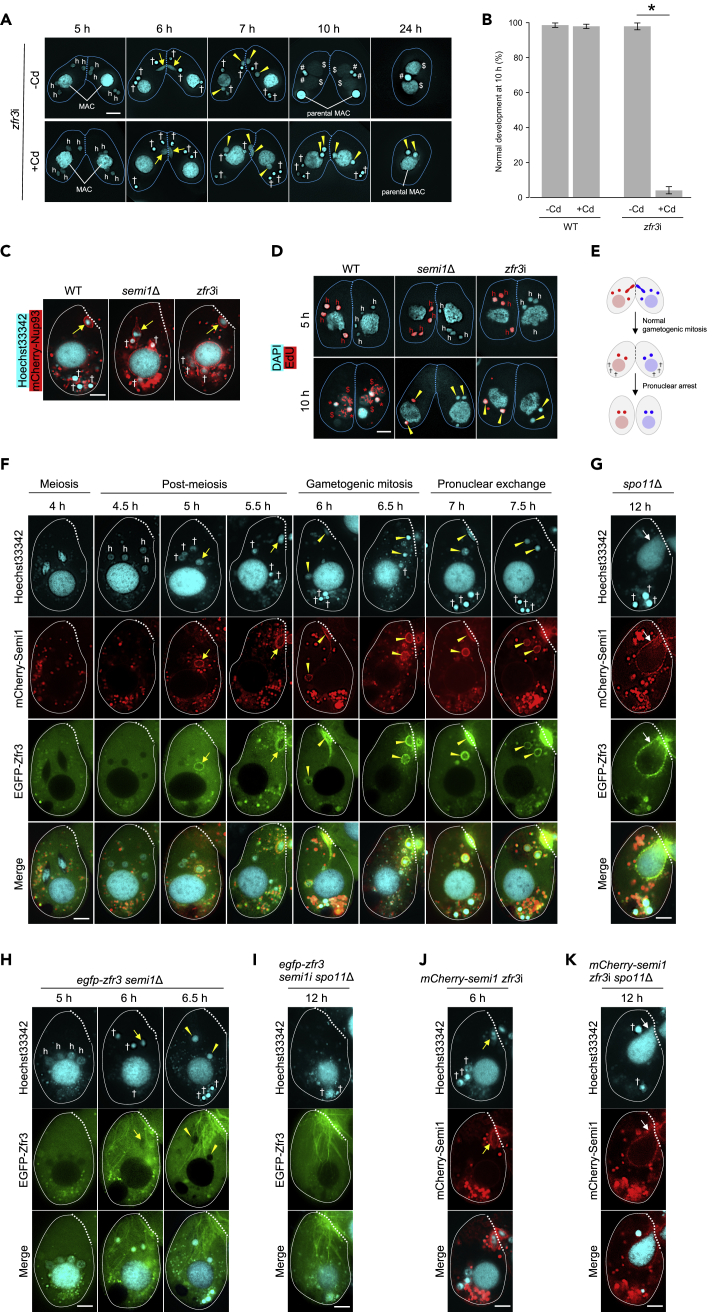
Zfr3-mediated Gametic Pronuclear Exchange Is Dependent on SEMI1 (A) Conjugating *zfr3*i cells (see also Figure S5) stained with DAPI. Top row, uninduced cells; bottom row, *zfr3* RNAi induced by CdCl_2_. (B) Percentage of cells with normal development of progeny nuclei at 10 h after the initiation of conjugation. Columns and error bars represent the means and standard deviations of three independent experiments. Asterisk (*) shows a significant difference (p < 0.01, as calculated by Tukey's HSD test on RStudio). (C) *zfr3*i does not affect hMIC attachment to the conjugation junction. The rim of the selected hMIC was visualized by mCherry-Nup93. (D) EdU (red) was incorporated into the MIC (left cell) for monitoring MIC exchange. hMICs remain in the labeled cell at 5 h after the induction of meiosis. EdU labeling is seen in both cells at 10 h in the WT and is restricted to the labeled cell in the *semi1*Δ and *zfr3*i genotypes. (E and F) (E) Pronuclear arrest phenotype of *zfr3*i cells. Different colors in the nuclei denote different genetic compositions as shown in Figure 1A. (F) Localization of Zfr3 in WT cells. (G) Localization of Zfr3 in *spo11*Δ cells. White arrow: MAC elongating toward the conjugation junction. (H) Zfr3 does not localize to the selected hMIC in *semi1*Δ cells. (I) Zfr3 does not localize to the MAC in *spo11*Δ *semi1*i cells. (J) Semi1 localizes to the selected hMIC in *zfr3*i cells. (K) Semi1 localizes to the MAC in *spo11*Δ *zfr3*i cells. (F–K) Bottom: merged image. h: hMIC; yellow arrow: selected hMIC; †: degenerating unselected hMIC; arrowhead: gametic pronucleus; $: progeny MAC; #: progeny MIC; dotted line: conjugation junction. Scale bar: 10 μm.

In the non-induced state, *zfr3*i mating cells underwent conjugation normally and completed the process as exconjugants (Figure 5A). In contrast, in the induced state, most *zfr3*i cells were di-micronuclear single cells and retained the parental MAC (Figures 5A and 5B), similar to the *semi1*Δ mutant (Figure 1A). Further, four hMICs were formed (Figure 5A), showing that meiosis was normal. Moreover, the markers of DNA repair (i.e. the disappearance of γH2AX foci and concomitant H3K56 acetylation and DNAPKcs and Rad51 localization) were seen in only one of the hMICs (Figures S5C–S5E), indicating that Zfr3 is not involved in hMIC selection. However, unlike in *semi1*Δ cells, gametogenic mitosis occurred in close proximity to the conjugation junction at 6 h after the initiation of conjugation (Figure 5A). Indeed, visualization of the nuclear rim with mCherry-tagged Nup93 (mCherry-Nup93; Figure S5G) clearly showed the selected hMIC attached to the conjugation junction in *zfr3*i cells (Figure 5C). These results suggest that loss of Zfr3 may affect either the exchange or the karyogamy of gametic pronuclei. To address this question, we labeled the MIC in cells of one mating type with 5-ethynyl-2′-deoxyuridine (EdU) prior to conjugation (Figure 5D). In the WT control, EdU was present in progeny MACs and MICs of both mating cells at 10 h due to pronuclear exchange followed by karyogamy (Figure 5D). In contrast, in *zfr3*i mating cells, EdU-incorporated DNA remained in the gametic pronuclei of cells of the original mating type (Figure 5D). We therefore conclude that Zfr3 is required for gametic pronuclear exchange (Figure 5E). As expected, pronuclear exchange did not take place in *semi1*Δ mating cells (Figure 5D).

**Figure 6:**
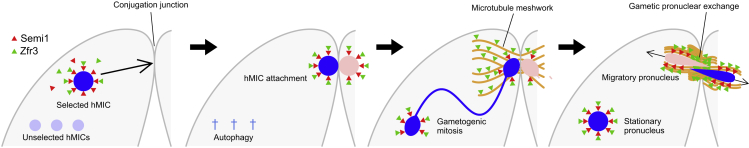
Model of Semi1 Recruitment of the Selected hMIC to the Conjugation Junction Once an hMIC is selected at random (see also Figure S6), Semi1 binds to its rim. Semi1 recruits the selected hMIC to the conjugation junction to enable hMIC attachment to occur in a microtubule-independent manner. Semi1 also recruits Zfr3 to the surface of the selected hMIC. Zfr3 may interact with microtubules at the conjugation junction, and the Semi1–Zfr3 complex is responsible for pronuclear exchange.

To analyze the subcellular localization of Zfr3, a strain expressing EGFP-tagged protein (EGFP-Zfr3) was created (Figure S5F) and mated with a strain expressing mCherry-Semi1. We found that EGFP-Zfr3 began to localize to the periphery of the selected hMIC simultaneously with mCherry-Semi1 (Figure 5F), whereas in *spo11*Δ mating cells, both fusion proteins co-localized at the periphery of the elongating MAC (Figure 5G). During pronuclear exchange, EGFP-Zfr3 strongly accumulated at the conjugation junction (Figure 5F), reflecting the likely role of Zfr3 in the exchange process. In the absence of Semi1 (by *semi1*i expression or in the *spo11*Δ background), EGFP-Zfr3 localized to neither the selected hMIC or MAC nor the conjugation junction, but instead formed numerous fibrous structures near to the conjugation junction (Figures 5H and 5I). In contrast, in the absence of Zfr3, mCherry-Semi1 localized normally to the selected hMIC in the WT background (Figure 5J) and to MAC in the *spo11*Δ background (Figure 5K). These results indicate that Semi1 is critical for both the correct localization and function of Zfr3. The fact that Zfr3 formed fibrous structures in the absence of Semi1 (Figures 5H and 5I) suggests that Zfr3 may be able to bind to the protein meshwork formed around the conjugation junction (Numata et al., 1985, Orias et al., 1983, Takagi et al., 1991) to promote gametic pronuclear exchange. Overall, these results show that Semi1 is essential for recruiting the selected hMIC to the conjugation junction to enable gametic pronuclear exchange (Figure 6).

## Discussion

### hMIC Selection Is Independent of Attachment to the Conjugation Junction

Different ciliate species contain differing numbers of MICs (from one to ∼20) and, hence, differing numbers of hMICs (from four to ∼80) following meiosis (Prescott, 1994). However, regardless of the number of hMICs formed, only one becomes the gametic pronucleus (Prescott, 1994). In addition, only one hMIC is selected in di- or tri-micronucleate *T*. *thermophila* mutants even though eight or 12 hMICs are generated by meiosis within a single cell (Nanney, 1953), suggesting that ciliates have the capability to ensure that only one hMIC undergoes post-meiotic development. The mechanism and reason for selecting a single hMIC from the meiotic products is unknown, but it may be important to cull the extra hMICs to prevent undesirable self-fertilization. The selection mechanism was previously assumed to target the hMIC located closest to or in contact with the conjugation junction after the second meiotic division (Gaertig and Fleury, 1992, Ishida et al., 1999, Numata et al., 1985, Takagi et al., 1991). This principle would resemble that of female meiosis in most flowering plants, in which only the meiotic product most proximal to the longitudinal axis of the ovule primordium is selected to become the functional megaspore (Demesa-Arevalo and Vielle-Calzada, 2013). However, the *semi1* mutation showed that in *T*. *thermophila* an hMIC that is not associated with the conjugation junction can be selected (Figure 3): on the contrary, hMIC attachment follows hMIC selection. Because MIC chromatin is symmetrically segregated to the hMICs during meiosis (Howard-Till and Loidl, 2018), (epi)genetic or physiological inequality between the meiotic products is unlikely to determine their different fates. This contrasts with female meiosis in animals, where asymmetric spindle formation produces big egg cells and small polar bodies (Clift and Schuh, 2013, Kursel and Malik, 2018).

In the absence of clear differences in the positions of hMICs (Figures 3D–3F), their different fates in WT cells may be due to a random process in which the arrival of the first hMIC in a suitable cellular location for selection to form a gametic pronucleus commits the other hMICs to autophagy. This situation resembles random X inactivation in female mammals, in which a signal from the active X chromosome (whose inactivation is prevented by blocking factors [Nicodemi and Prisco, 2007]) inactivates the other X chromosome (or chromosomes in triple-X cells) (Lu et al., 2017, Pollex and Heard, 2019). In this case, the inactivating signal is not a protein, which would have to be expressed by one of the X chromosomes and then translated and imported into the nucleus, where it would affect both X chromosomes similarly. Instead, the message may consist of non-coding RNA (Pollex and Heard, 2019). Although in *Tetrahymena* the source and recipient of the signal are not different chromosomes within a nucleus but different nuclei within a cell, we speculate that a similar principle could work in hMIC selection.

### Semi1 Positions the Selected hMIC at the Conjugation Junction

Several lines of evidence indicate that microtubules form a meshwork around the conjugation junction to promote gametic pronuclear exchange (Orias et al., 1983, Kushida et al., 2015). This meshwork is also thought to be responsible for hMIC attachment to the conjugation junction by trapping the selected hMIC (Gaertig and Fleury, 1992). In addition to microtubules, a filament-forming citrate synthase, Cit1, also forms a meshwork around the conjugation junction (Numata et al., 1985, Takagi et al., 1991). Therefore, it is possible that Semi1 might utilize the polymerization or depolymerization forces of tubulin or Cit1 to move the selected hMIC toward the conjugation junction. Alternatively, Semi1 may interact with motor proteins or Rab GTPases to drive the movement of the selected hMIC along these filaments. However, contrary to our expectations, none of these proteins co-precipitated with Semi1 (Tables 1 and S1). This result suggests that Semi1-mediated hMIC attachment to the conjugation junction may bear little (or no) resemblance to the cytoskeleton-dependent nuclear positioning or membrane trafficking that occurs in other organisms (Tran et al., 2001, Gundersen and Worman, 2013, Huelsmann and Brown, 2014, Kiral et al., 2018). It is also possible that Semi1 has a regulatory rather than an active role in cytoskeleton-dependent nuclear relocation.

Alternatively, the molecular affinity between Semi1 and proteins expressed at the conjugation junction may be sufficient to trap the selected hMIC. A previous proteomic analysis of isolated junctions from conjugating cells identified 15 proteins, including an epiplasmic protein (Cole et al., 2008). In addition, recent studies identified a fusogen protein Hap2, a homolog of male-gamete-specific protein (Cole et al., 2014, Pinello et al., 2017), and a zinc finger protein Zfr1 (Xu et al., 2012), both of which are essential components of the conjugation junction. One of the 15 proteins, Ftt18, was originally discovered as a basal body component (Kilburn et al., 2007). It was also identified as a Semi1 interactor in our experiments (Table S1) and localized to both the conjugation junction and the periphery of the selected hMIC (Figure S4). Unfortunately, the mutant phenotype could not be studied owing to technical issues. However, it remains possible that interaction (direct or indirect) between Semi1 and Ftt18 is sufficient to bind an hMIC to the conjugation junction once it comes into close proximity. This suggests a model for hMIC selection in which all hMICs have the potential to pass close to the conjugation junction by random movement, but only a nucleus with the appropriate membrane makeup (the selected hMIC or in some cases the MAC) would become trapped there.

### Markers on the Nuclear Surface May Determine the hMIC Position

A hMIC may acquire some similar properties to the MAC upon nuclear fate determination (Figures 4A–4C), which may guide Semi1 to the periphery of selected hMIC to ensure hMIC attachment. In fact, in the selected hMIC histone H3 is acetylated at several lysine residues (Akematsu et al., 2017) other than K56 (Figure 3A). Acetylation of histone H3 at these sites is strongly enriched in euchromatin (Wang et al., 2008, Tie et al., 2009) and also characteristic of the active MAC (Chicoine and Allis, 1986, Pfeffer et al., 1989). This change may also be critical to protect the selected hMIC from autophagy, which eliminates the unselected hMICs (Liu and Yao, 2012). The first hMICs to come into contact with the MAC may undergo changes that cause its membrane properties to resemble those of the MAC, which leads to changes in the chromatin, resulting in selection. We showed that perinuclear Semi1 contributes to nuclear migration toward the conjugation junction (Figures 2A and 4A), probably without a direct interaction with microtubules or motor proteins (Table 1). Similar functional relationships between surface markers and the characteristic internuclear mobility may be general features of mating *T*. *thermophila* cells. For instance, the degenerating parental MAC migrates toward the posterior of the cell (Cole and Sugai, 2012). The surface of this nucleus is decorated with glycocalyx compounds and phosphatidylserine, which are absent from the other nuclei within the cell, and may be recognized by the autophagic machinery (Akematsu et al., 2010). Similarly, the unselected hMICs migrate to the posterior region of the cell (Cole and Sugai, 2012). Although the direct relevance of this surface property to nuclear migration is unknown, loss of autophagy-related genes prevents migration and, hence, lysosomal acidification of the nucleus (Liu and Yao, 2012, Akematsu et al., 2014). Different nuclear surface molecules may therefore be recognized by different intracellular trafficking pathways so as to guide the different nuclei to specific cell compartments where they are differentially processed.

### Limitations of the Study

Owing to limited experimental conditions and equipment, we were unable to perform time-lapse imaging to capture hMIC selection in live cells. Also, topology of Semi1 in the nuclear membrane is unclear because a reliable topology prediction tool is currently unavailable for the nuclear envelope proteins.

## Methods

All methods can be found in the accompanying Transparent Methods supplemental file.

## Supplemental Information

Document S1. Transparent Methods, Figures S1–S5, and Table S1
