# Supplementary material for: The Transmembrane Protein Semi1 Positions Gamete Nuclei for Reciprocal Fertilization in Tetrahymena
Source: iScience. 2019 Nov 28;23(1):100749. doi: 10.1016/j.isci.2019.100749 (PMC6941865; doi:10.1016/j.isci.2019.100749)
Supplement: Document S1. Transparent Methods, Figures S1–S5, and Table S1 [file mmc1.pdf]

ISCI, Volume *23*

Supplemental Information

The Transmembrane Protein Semi1 Positions Gamete Nuclei for Reciprocal Fertilization in *Tetrahymena*

Takahiko Akematsu, Rosalía Sánchez-Fernández, Felix Kosta, Elisabeth Holzer, and Josef Loidl

Supplemental information

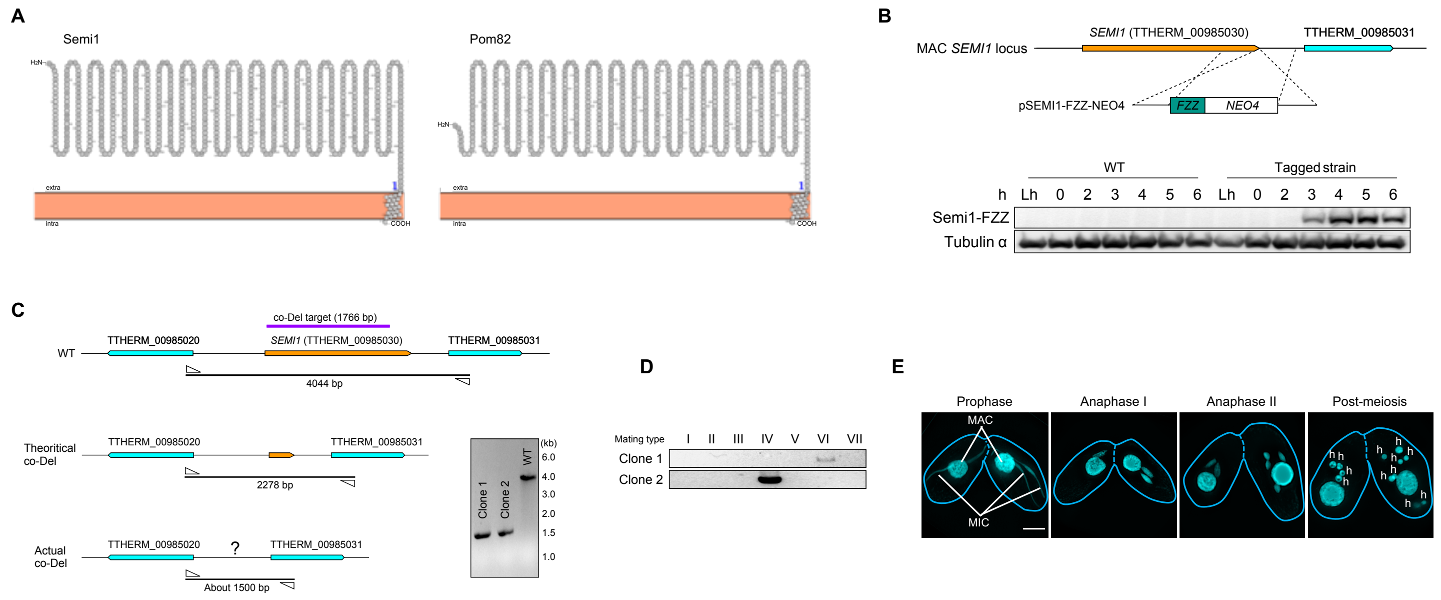

Figure S1. Characterization of Semi1 and generation of somatic semi1 knockout mutants. Related to Figure 1.

(A) Possible topologies of the Semi1 (left) and the Pom82 (right) proteins in the MIC envelope. Pom82 is a transmembrane nucleoporin present in the MIC (Iwamoto et al., 2017). Since topology prediction tools specialized for nuclear envelope proteins do not exist, we used PROTTER (http://wlab.ethz.ch/protter/start/), an open source tool for visualization of general transmembrane topology, to predict Semi1's orientation in the MIC envelope. The orange bars represent cell membrane. As seen in Pom82, the C-terminal and the other regions of Semi1 were predicted to be a transmembrane domain and to face extracellular space, respectively. This result suggests that the non-transmembrane region of Semi1 is exposed to the cytosol as of Pom82. (B) Construction of FZZ-tagged Semi1-expressing cells. The pSEMI1-FZZ-NEO4 plasmid, containing an FZZ tag and neomycin resistance cassette (*NEO4*), was integrated into the MAC *SEMI1* locus by homologous recombination. Conjugation-specific expression of FZZ-tagged Semi1 was confirmed by western blotting. Cells were collected at the indicated times after the initiation of conjugation. Lh: exponentially growing cells; 0: starved cells. Tubulin α was the loading control. (C) Generation of *semi1*Δ cells and PCR confirmation. PCR primers are indicated by white triangles. The WT *SEMI1* genomic locus (1766 bp, indicated by purple line) was targeted by the co-Del plasmid. A DNA fragment of 2278 bp is expected from co-Del cells. However, fragments were about 1500 bp, indicating deletion of flanking regions. (D) Mating types of the *semi1*Δ clones were determined by PCR using mating-type-specific primer sets (gift from Dr Marcella D. Cervantes, Albion College, MI, USA). (E) Induction of meiosis in the unexchanged gametic pronuclei of *semi1* Δ exconjugant (see also Figure 1B). A *semi1* Δ exconjugant (right) mated with a parental *semi1* Δ cell (left) was stained with DAPI. The unexchanged gametic pronuclei in right cell became eight meiotic products, the number of which is twice as many as the left cell, suggesting that *semi1* Δ exconjugants undergo DNA endoreplication in the unexchanged gametic pronuclei prior to the next round of meiosis. h: hMICs. Dotted line: conjugation junction. Scale bar: 10 μm.

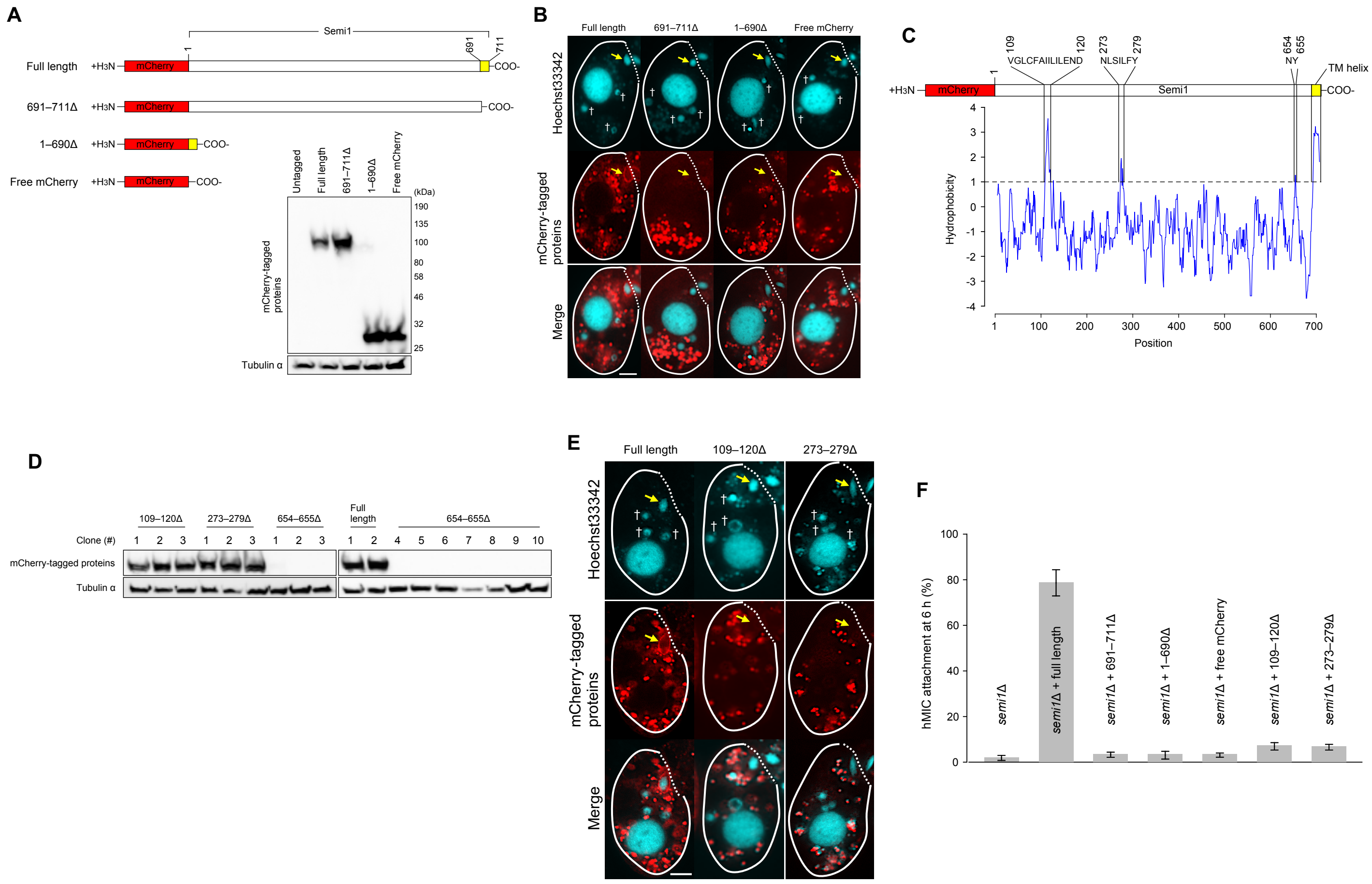

Figure S2. Perinuclear localization of Semi1 mediates hMIC attachment to the conjugation junction. Related to Figure 1.

(A) Schematic representation of full-length mCherry-Semi1 and truncated variants. The yellow box represents the transmembrane helix. Western blot was used to confirm protein expression. Tubulin α was the loading control. (B) Truncated mCherry-Semi1 variants do not localize to the perinuclear region. Hoechst 33342 staining shows the position of the MAC and hMICs in living cells was. Arrowhead: selected hMIC; †: degenerating unselected hMIC. Dotted line: conjugation junction. Scale bar: 10 μm. (C) A schematic diagram showing the hydrophobic regions of Semi1, as profiled by ProtScale (https://web.expasy.org/protscale/). The three non-transmembrane regions (109–120, 273–279, and 654–655) were deleted from the mCherry-Semi1 expression construct. TM: transmembrane. (D) Western blotting analysis of the expression of mCherry-Semi1 deletion variants. Expression of variant 654–655Δ was undetectable in 10 different clones, indicating that residues N654 and Y655 are important for protein stability. (E) mCherry-Semi1 variants lacking the transmembrane region fail to localize to the perinuclear region of the selected hMIC. Arrowhead: selected hMIC; †: degenerating unselected hMIC. Dotted line: conjugation junction. Scale bar: 10 μm. (F) Percentage of cells with normal hMIC attachment to the conjugation junction at 6 h after the initiation of conjugation. The truncated variants and the deleted versions of mCherry-Semi1 were expressed in *semi1*Δ cells. Columns and error bars represent means and standard deviations of three measurements.

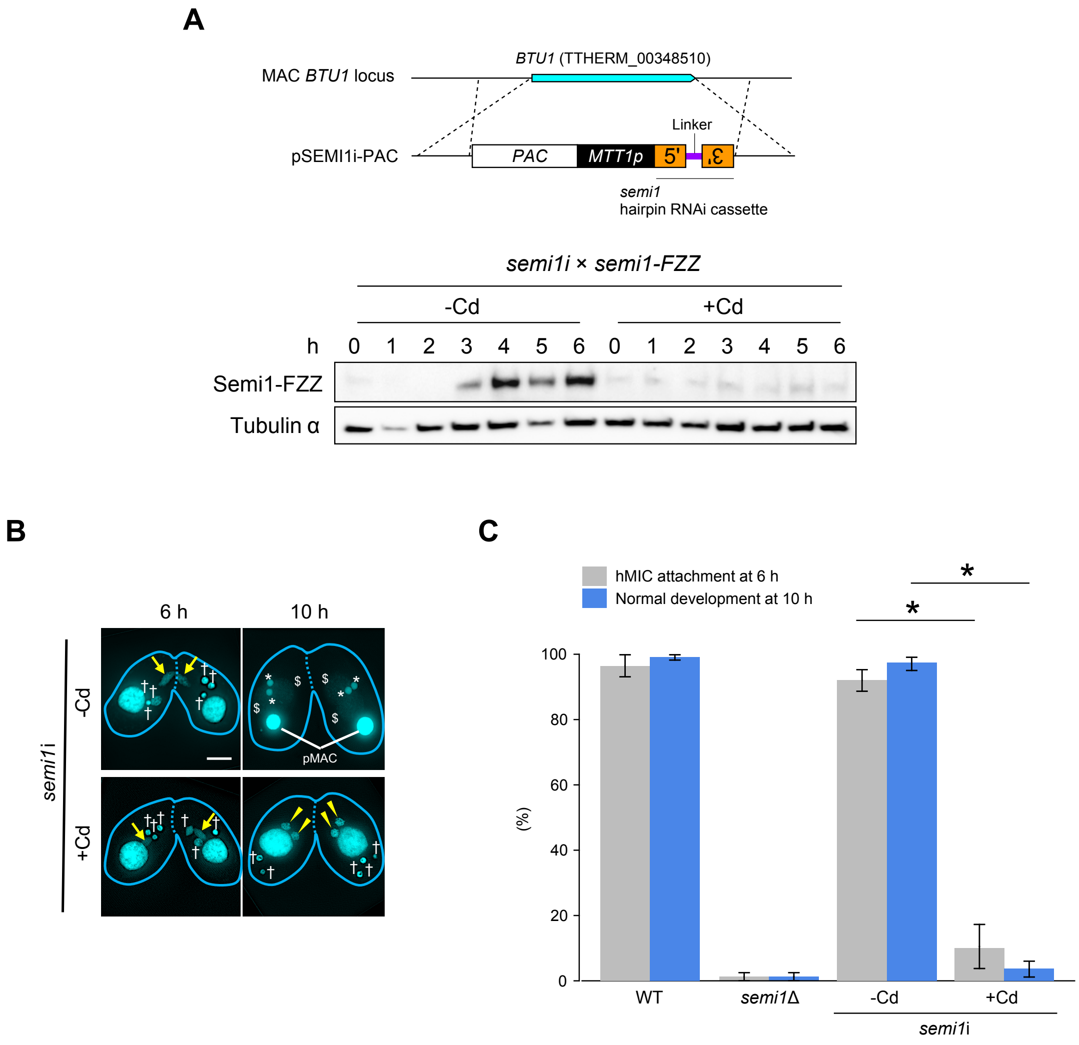

Figure S3. *semi1* RNAi expression has the same phenotype as the *semi1*Δ mutant. Related to Figure 4.

(A) Generation of *semi1*i-expressing cells. The pSEMI1i-PAC plasmid, containing a puromycin resistance marker (*PAC*), cadmium-inducible *MTT1* promoter, and hairpin RNAi cassette, was integrated into the MAC *BTU1* locus of Semi1-FZZ-expressing cells by homologous recombination. Western blotting confirms that Semi1-FZZ expression is lost following semi1 RNAi induction. Tubulin α was the loading control. (B) Cells expressing *semi1*i had the same phenotype as *semi1*Δ cells at 6 h and 10 h after the expression of RNAi. Arrow: selected hMIC undergoing gametogenic mitosis; †: degenerating unselected hMIC; arrowhead: gametic pronucleus; $: progeny MAC; #: progeny MIC. Dotted line: conjugation junction. Scale bar: 10 μm. (C) Percentage of cells with normal hMIC attachment at 6 h after the initiation of conjugation and with normal development of progeny nuclei at 10 h. Columns and error bars represent the means and standard deviations of three independent experiments. Asterisk (*) shows a significant differences between columns (p<0.01, as calculated by Tukey's HSD test on RStudio).

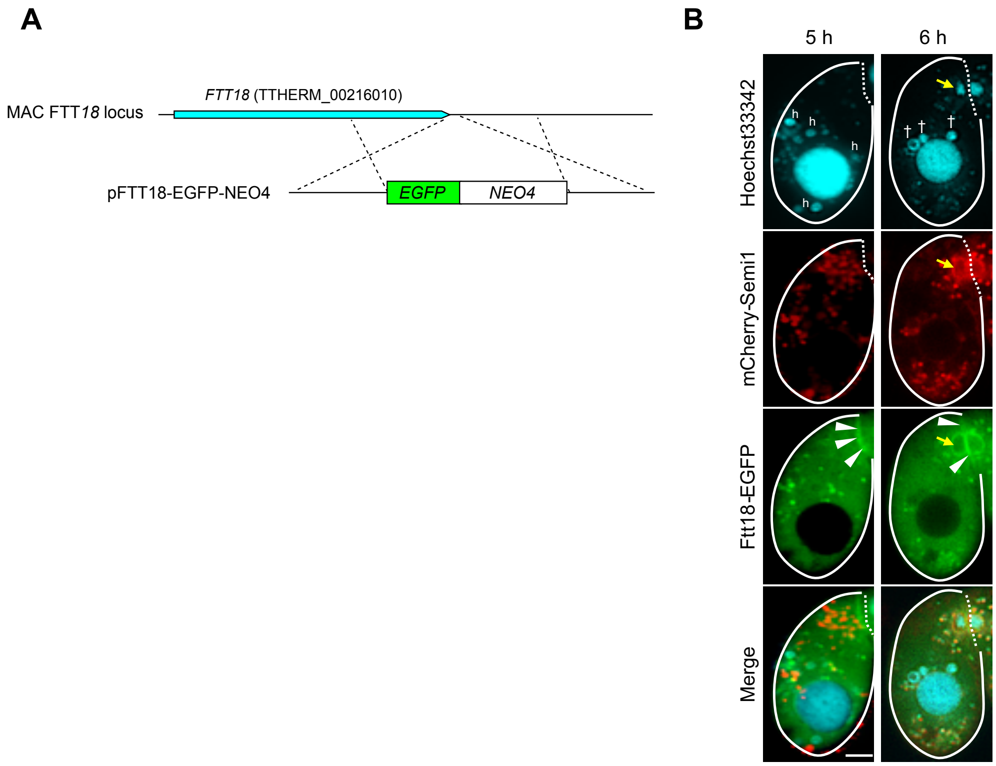

Figure S4. Ftt18 localizes to both the conjugation junction and the selected hMIC. Related to Table 1.

(A) Generation of cells expressing EGFP-tagged Ftt18. The pFTT18-EGFP-NEO4 plasmid, containing an *EGFP* tag and neomycin resistance cassette (*NEO4*), was integrated into the MAC *FTT18* locus by homologous recombination. (B) Ftt18-EGFP (arrowheads) localizes to the conjugation junction at 5 h after the initiation of conjugation and co-localizes with mCherry-Semi1 to the selected hMIC (arrow). h: hMIC; †: degenerating unselected hMIC. Dotted line: conjugation junction. Scale bar: 10 μm.

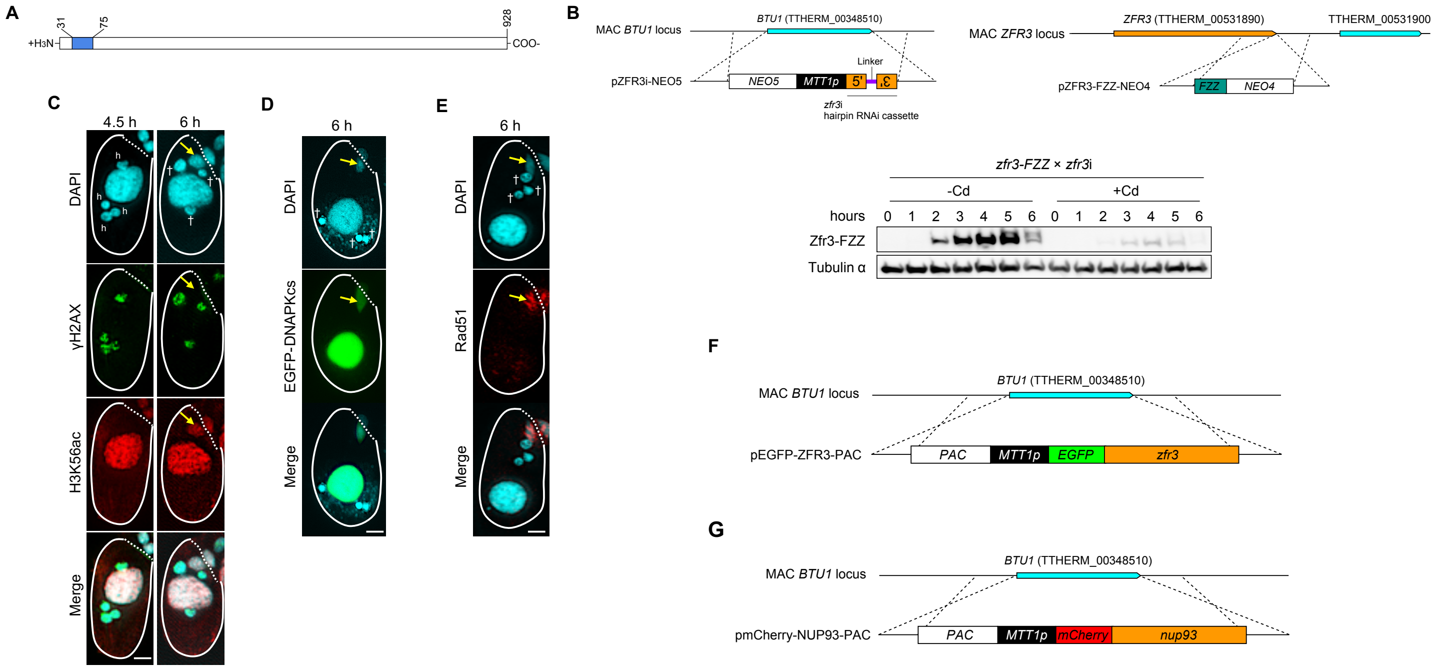

Figure S5. RNAi-mediated knockdown of the Zfr3 conjugation-specific protein. Related to Figure 5.

(A) Primary structure of the Zfr3 protein. No homolog has been found in other organisms. The blue box represents a C3HC4 type zinc finger domain. (B) Generation of cell lines expressing a *zfr3* RNAi (*zfr3i*) (upper left) and FZZ-tagged Zfr3 (upper right). The pZFR3i-NEO5 plasmid, containing a paromomycin resistance marker (*NEO5*), cadmium-inducible *MTT1* promoter, and hairpin RNAi cassette, or the pZFR3-FZZ-NEO4 plasmid, containing a FZZ tag and neomycin resistance cassette (*NEO4*), were integrated into the MAC *BTU1* locus or the MAC *ZFR3* locus, respectively, by homologous recombination. The resulting cell lines were mated, and protein was extracted. Western blotting showed that Zfr3-FZZ expression is strongly reduced upon *zfr3i* induction. Tubulin α was the loading control. (C) γH2AX localizes to all four hMICs at 4.5 h after the initiation of conjugation in *zfr3*i cells. At 6 h, one hMIC (arrow) loses γH2AX staining concomitant with histone H3 acetylation at lysine 56 (H3K56ac). h: hMIC; †: degenerating unselected hMIC. Dotted line: conjugation junction. Scale bars: 10 μm. (D) EGFP-tagged DNAPKcs localizes to the hMIC nearest to the conjugation junction (arrow) in *zfr3*i cells at 6 h after the initiation of conjugation. †: degenerating unselected hMIC. Dotted line: conjugation junction. Scale bars: 10 μm. (E) Rad51 localizes to the hMIC nearest to the conjugation junction (arrow) in *zfr3*i cells. †: degenerating unselected hMIC. Dotted line: conjugation junction. Scale bars: 10 μm. (F) Generation of a cell line expressing EGFP-tagged Zfr3. The pEGFP-ZFR3-PAC plasmid, containing a puromycin resistance marker (*PAC*), cadmium-inducible *MTT1* promoter, and EGFP-Zfr3 expression cassette, was integrated into the MAC *BTU1* locus by homologous recombination. (G) Generation of a cell line expressing mCherry-tagged NUP93. The pmCherry-ZFR-PAC plasmid, containing a puromycin resistance marker (*PAC*), cadmium-inducible *MTT1* promoter, and EGFP-Zfr3 expression cassette, was integrated into the MAC *BTU1* locus by homologous recombination.

Table S1. MS identification of constitutively expressed interaction partners of Semi1. Related to Table 1.

Gene ID (TTHERM_)	AvgCount	Control count	p-value	Protein name	Description
00216010	133.5	0|4	0	Ftt18	14-3-3 protein
00194540	81	4|6	0	None	TATA-binding protein interacting (TIP20) protein
00105110	61	7|12	0	Hsp70	HSP70 heat shock 70 kDa protein
00158520	43.5	3|2	0	Hsp82	HSP82 predicted protein
00161720	41	0|0	0	None	Zinc finger in N-recognin family protein
00579319	35.5	0|0	0	None	Hypothetical protein
00160770	35	0|1	0	Ftt49	14-3-3 protein
00535500	32	0|1	0	None	DnaJ domain protein
00047040	29	0|0	0	None	Ubiquitin carboxy-terminal hydrolase
00550700	25.5	0|2	0	None	Importin protein
00299570	24.5	0|3	0	None	Pyridine nucleotide-disulfide oxidoreductase
00865270	24.5	1|3	0	Ydj1	DnaJ carboxy-terminal domain protein
000522989	21	0|0	0	None	Hypothetical protein
00339610	20	1|4	0	Rpn1	RPN1 26S proteasome regulatory subunit
00444670	20	1|0	0	Hsp90	HSC82 heat shock protein HSP90
00476820	20	0|0	0	Rvb1	RVB holliday junction ATP-dependent DNA helicase RuvB
00138370	17.5	0|2	0	Atg7	ATG7 ubiquitin-like modifier-activating enzyme
00471950	17.5	1|0	0	None	Hypothetical protein
00891190	17.5	0|1	0	None	Hypothetical protein
00780580	17	0|5	0	Cyc16	CYC16 amine-terminal domain cyclin
00049030	16.5	0|0	0	None	Na,H/K antiporter P-type ATPase, alpha subunit family protein
00591660	16.5	0|0	0	None	Importin-beta amine-terminal domain protein
00502340	15.5	0|0	0	None	Glycerol-3-phosphatase 0-acyltransferase
001014659	15.5	0|5	0.01	None	26S proteasome regulatory complex ATPase RPT2
00068110	15.5	1|6	0.03	Rpt3	RPT3 26S protease regulatory subunit 6B
00627000	14.5	0|0	0	None	SIT4 Phosphatase-associated protein
00372460	14	0|0	0	Flp10	Phospholipid-translocating P-type ATPase, flippase family protein
00426310	14	0|3	0	None	Chaperone DnaJ
00771980	13	0|1	0	None	Tetratricopeptide repeat protein
00444500	12	0|0	0	Gcn1	HEAT repeat protein
00856430	12	0|0	0	None	E1-E2 ATPase family protein
00279670	12	0|4	0.01	Rpt1	RPT1 26S proteasome regulatory subunit
00158000	10	0|0	0	None	Transmembrane protein putative
00354810	10	0|0	0	None	Acyltransferase
00313530	10	0|3	0.01	None	Transmembrane protein putative
00657230	9	0|1	0	None	Hypothetical protein
00191240	9	1|4	0.02	Rpn7	RPN7 26S proteasome non-ATPase regulatory subunit 6
00578940	9	0|4	0.04	Rpn5	RPN5 26S proteasome non-ATPase regulatory subunit
01049200	9	0|4	0.04	None	Glutamate/leucine/phenylalanine/valine dehydrogenase
00011220	8.5	0|1	0	None	Kinase domain protein
00703480	8	0|0	0	None	Transmembrane protein putative
01084370	8	0|0	0	Tpa8	TPA8 sarco/endoplasmic reticulum calcium-translocating P-type ATPase
00047110	7	0|1	0	Alg5	ALG5 dolichyl-phosphate beta-glucosyltransferase
00437670	6.5	0|0	0	None	DnaJ domain protein

Transparent Methods

Culture methods and the induction of cell mating (conjugation)

WT *T. thermophila* strains CU428.2 (mating type VII, RRID:TSC_SD00178) and B2086 (mating type II, RRID:TSC_SD01627) were obtained from the *Tetrahymena* Stock Center, Cornell University (http://tetrahymena.vet.cornell.edu/). The GFP-Nup93-expressing strain (Iwamoto et al., 2009) was a gift from Dr Masaaki Iwamoto (Advanced ICT Research Institute, Kobe, Japan). Strains expressing EGFP-DNAPKcs (Akematsu et al., 2017) and *spo11*Δ (Mochizuki et al., 2008) were constructed previously. Cells were grown at 30°C in super proteose peptone (SPP) medium containing 1% proteose peptone (Becton Dickinson, Sparks, MD, USA), 0.1% yeast extract (Becton Dickinson), 0.2% glucose (Sigma-Aldrich, St. Louis, MO, USA), and 0.003% EDTA-Fe (Sigma-Aldrich). To make them competent for mating, cells at mid-log phase (approximately 10^6 cells/mL) were washed with 10 mM Tris-HCl (pH7.4), resuspended in 10 mM Tris-HCl (pH7.4), and starved at 30°C for ~16 h. To induce mating, equal numbers of cells of two different mating types were mixed together and incubated at 30°C.

semi1 gene disruption

A 1776-bp fragment of the *SEMI1* open reading frame (ORF) was amplified from CU428.2 genomic DNA using PrimeSTAR Max DNA polymerase (TaKaRa, Kusatsu, Japan, Cat. R045A) and primers #1 and #2 (see below). The amplified fragment was cloned into the NotI site of the pMcoDel plasmid (Hayashi and Mochizuki, 2015) using the NEBuilder HF DNA Assembly kit (New England Biolabs, Ipswich, MA, USA, Cat. E5520S). NEB 5-alpha competent *E. coli* cells (New England Biolabs, Cat. C29871) were used to amplify all plasmids created in this study. These plasmids were used for biolistic transformation (Cassidy-Hanley et al., 1997), and 100 μg/mL paromomycin sulfate (Sigma-Aldrich, Cat. P8692-25G) was used to select the transformants. Deletion of the target locus from the MAC was confirmed using the primer set #3 and #4 (see below).

#	Primer name	Sequence (5′→3′)
1	semi1_co-del_F	CTTTATTGTTATCATCTTATGACCGCGGATTTTACTTAATTGATTGGCAC
2	semi1_co-del_R	CTCATCAAGTTGTAATGCTAAAATGCTTGTCATAGGATTACATTCACTAG
3	semi1_check_F	ATCCCAGAAGGATCCAAC
4	semi1_check_R	GTCAGTTTAGTCACGAGC

RNAi vector construction and gene knockdown

Target sequences used in hairpin RNA constructs (486 bp of the SEMI1 ORF and 500 bp of the *ZFR3* ORF) were amplified from CU428.2 genomic DNA using PrimeSTAR Max DNA polymerase and the following primer sets: #5 and #6 for the *SEMI1* forward fragment, #7 and #8 for the *SEMI1* reverse fragment, #9 and #10 for the *ZFR3* forward fragment, and #11 and #12 for the *ZFR3* reverse fragment (see below). Amplified forward and reverse target fragments were cloned into the BamHI–BamHI and PstI–PstI sites, respectively, of pAkRNAi-NEO5 (Akematsu et al., 2018) with the NEBuilder HF DNA Assembly kit. to create the hairpin cassette. For *semi1*, the *NEO5* cassette of the backbone plasmid, which confers paromomycin resistance (Mochizuki, 2008), was replaced by a puromycin resistance marker (*PAC*) (Iwamoto et al., 2014) under the *MTT2* copper-inducible promoter (Boldrin et al., 2008, Akematsu et al., 2017) using T4 DNA ligase (New England Biolabs, Cat. M0202S). The resulting plasmids (pSEMI1i-PAC and pZFR3i-NEO5) were linearized with SacI and KpnI (New England Biolabs) before biolistic transformation. The *PAC* cassette was activated by adding 630 μM $CuSO_4$ to the cells, with the addition of 200 μg/mL puromycin dihydrochloride (Cayman Chemical, Ann Arbor, MI, USA, Cat. CAYM13884-500) to select transformants. RNAi was induced in cells carrying the hairpin construct by adding 0.075 μg/mL $CdCl_2$ during pre-conjugation starvation to promote double stranded RNA expression from the *MTT1* cadmium-inducible promoter (Shang et al., 2002).

#	Primer name	Sequence (5′→3′)
5	semi1_RNAi_5F	TAAACTTAAACATCCCGGGGGATCCGCTAAACAAAAAGTGAGGGAAAGCC
6	semi1_RNAi_5R	TTGCATATCCGTTACTTACGGATCCTTCATGAGATGACTTTTGAGAGCTG
7	semi1_RNAi_3F	TAAAAGAAGAATTCAAAGGCTGCAGTTCATGAGATGACTTTTGAGAGCTG
8	semi1_RNAi_3R	GCTGACCGATTCAGTTCGCCTGCAGGCTAAACAAAAAGTGAGGGAAAGCC
9	zfr3_RNAi_5F	TAAACTTAAACATCCCGGGGGATCCCACTTTGAAGAGACATAATCTTCAG
10	zfr3_RNAi_5R	TTGCATATCCGTTACTTACGGATCCCTGTTAGAGTCATCATTATTAGAATATCTAG
11	zfr3_RNAi_3F	TAAAAGAAGAATTCAAAGGCTGCAGCTGTTAGAGTCATCATTATTAGAATATCTAG
12	zfr3_RNAi_3R	GCTGACCGATTCAGTTCGCCTGCAGCACTTTGAAGAGACATAATCTTCAG

C-terminal epitope tagging

For the expression of Semi1-FZZ (composed of 3× FLAG, a TEV protease-cleavage site, and ZZ domain of protein A (Lee and Collins, 2007), Ftt18-EGFP, and Zfr3-FZZ, C-terminal tagging of endogenous proteins was done using a knock-in strategy (Kataoka et al., 2010). In short, approximately 1 kb from the 3′ end of the coding sequence and 1 kb from a downstream region were amplified with PrimeSTAR Max DNA polymerase and the following primer sets: *SEMI1*, #13 and #14 for the C-terminus and #15 and #16 for the downstream region; *FTT18*, #17 and #18 for the C-terminus and #19 and #20 for the downstream region; and *ZFR3*, #21 and #22 for the C-terminus and #23 and #24 for the downstream region (see below). Amplified fragments were cloned into the pFZZ-NEO4 (GenBank: AB570112.1) or pEGFP-NEO4 (GenBank: AB570109.1) plasmid using the NEBuilder HF DNA Assembly kit or T4 DNA ligase. The resulting plasmids (pSEMI1-FZZ-NEO4, pFTT18-EGFP-NEO4, and pZFR3-FZZ-NEO4) were linearized by digestion with SacI and KpnI before biolistic transformation of *T. thermophila* cells. The *NEO4* cassette was activated by adding 1 μg/mL $CdCl_2$ to the cells, with 100 μg/mL paromomycin sulfate (Sigma-Aldrich) used to select the transformants.

#	Primer name	Sequence (5′→3′)
13	semi1_Ctag_5F	AAAGGGAACAAAAGCTGGAGCTCGACATTCAATGGACTGTTTGGGAGA
14	semi1_Ctag_5R	TCATGATCTTTGTAATCGGATCCATAATAAAATATTAGTAAAAAATAACAAAATAGCAAC
15	semi1_Ctag_3F	AGTCCTCGAGGTTCAATAATGCATAGATAGTTACAACC
16	semi1_Ctag_3R	AGTCGGTACCTCCGGGTTAGATATCAACAATAGGTTAT
17	ftt18_Ctag_5F	CTAAAGGGAACAAAAGCTGGAGCTCGTAGTAAGCGGTCAACTACATTACT
18	ftt18_Ctag_5R	CTTCACCCTTAGAAACCATGGATCCTTCTTATTATTCTTCAGCATCGTCT
19	ftt18_Ctag_3F	GCTTATCGATACCGTCGACCTCGAGGAGATGGTGTGAAAATCAAACAAAA
20	ftt18_Ctag_3R	CTCACTATAGGGCGAATTGGGTACCGTGCTAATGAATTTCGCCAAATAGC
21	zfr3_Ctag_5F	AAAGGGAACAAAAGCTGGAGCTCGCGATGAAAAATCTGAAATCAAGAA
22	zfr3_Ctag_5R	AAGTTCTTCACCCTTAGAAACCATGGATCCATTTTGATTTAGTTTCAATAGCTTTTG
23	zfr3_Ctag_3F	GCTTATCGATACCGTCGACCTCGAGGAGAAATAATCTTTAATGTTAAACTTGAAATATCC
24	zfr3_Ctag_3R	CACTATAGGGCGAATTGGGTACCTTTGCGGAATCAGAAATTGATCTGC

N-terminal epitope tagging

The *SEMI1*, *ZFR3*, and NUP93 ORFs were amplified from CU428.2 genomic DNA with PrimeSTAR Max DNA polymerase and the following primer sets: #25 and #26 for the *SEMI1* ORF, #27 and #28 for the *ZFR3* ORF, and #29 and #30 for *NUP93* ORF (see below). Amplified fragments were cloned into the BamHI–SpeI sites of pBNMB1-EGFP (a gift from Dr. Kazufumi Mochizuki, Institute of Human Genetics, Montpellier,

France), which contains the *MTT1* promoter, *NEO5* cassette, and the 5′ and 3′ portions of the *BTU*1 genomic locus for homologous recombination, using the NEBuilder HF DNA Assembly kit. To transfect these plasmids into paromomycin-resistant mutant strains, we replaced the *NEO5* cassette in the plasmids with the *PAC* cassette, excised from pSEMI1i-PAC with SalI plus XmaI (New England Biolabs), using T4 DNA ligase. For Semi1 and Nup93 tagging, the *EGFP* cassette was replaced by the *mCherry* cassette, which was amplified from pmCherry-NEO4 (GenBank: AB570110.1) using PrimeSTAR Max DNA polymerase and primers #31 and #32 (see below). The resulting plasmids (pmCherry-SEMI1-PAC, pEGFP-ZFR3-NEO5, pEGFP-ZFR3-PAC, and pmCherry-NUP93-PAC) were linearized by digestion with SacI and KpnI before biolistic transformation. Protein expression was induced in cells by adding 0.075 μg/mL $CdCl_2$ to starved cells.

#	Primer name	Sequence (5′→3′)
25	semi1_Ntag_F	GGATGAATTATATAAGGGATCCATGGATTTTACTTAATTGATTGGCAC
26	semi1_Ntag_R	CGATTCAGTTCGCTCAACTAGTATAATAAAATATTAGTAAAAAATAACAAAATAGC
27	zfr3_Ntag_F	GGATGAATTATATAAGGGATCCATGCAACACTTTGAAGAGACATAATC
28	zfr3_Ntag_R	GACCGATTCAGTTCGCTCAACTAGTATTTTGATTTAGTTTCAATAGCTTTTG
29	Nup93_Ntag_F	GGATGAATTATATAAGGGATCCATGAGTTTTACTGTTGCTCGCGATG
30	Nup93_Ntag_R	CGATTCAGTTCGCTCAACTAGTAACTTAATCTGTAACTTAGGCATTG
31	mcherry_F	AAATAATAATACTAAACTTAAACATATGGTTTCAAAAGGAGAAGAAGATA
32	mcherry_R	CAAGTAAATGCTCTAACATGGATCCACTAGTTTTGTAAAGTTCATCCATA

Construction of strains expressing truncated mCherry-Semi1

The *SEMI1* ORF lacking the transmembrane portion (691–711Δ) was amplified from CU428.2 genomic DNA using PrimeSTAR Max DNA polymerase and primers #33 and #34 (see below). The amplified fragment was cloned into SpeI–SpeI sites of pmCherry-SEMI1-PAC using the NEBuilder HF DNA Assembly kit. The sequence encoding the cytoplasmic portion of Semi1 (1–690) was removed from pmCherry-SEMI1-PAC by inverse PCR using PrimeSTAR Max DNA polymerase and primers #35 and #36 (see below), followed by digestion with BamHI and self-ligation with T4 DNA ligase. A free mCherry expression plasmid was also created by digesting pmCherry-SEMI1-PAC with SpeI followed by self-ligation with T4 DNA ligase. The resulting plasmids (pmCherry-691–711Δ-PAC, pmCherry-1–690Δ-PAC, and pmCherry-PAC) were linearized with SacI and KpnI before biolistic transformation. Protein expression was induced in cells by adding 0.075 μg/mL $CdCl_2$ during starvation.

#	Primer name	Sequence (5′→3′)
33	semi1_691–711Δ_F	TATGGATGAACTTTACAAAACTAGTATGGATTTTACTTAATTGATTGGCA
34	semi1_691–711Δ_R	GACCGATTCAGTTCGCTCAACTAGTATCTTTTTTTGAGTAATTGCTCTTTTC
35	semi1_1–690Δ_F	AGTCGGATCCATAGTGACAATAACTTTTTTAATTTTGTTGCTATTTTG
36	semi1_1–690Δ_R	AGTCGGATCCACTAGTTTTGTAAAGTTCATCCATA

Construction of strains expressing mutated mCherry-Semi1

Inverse PCR was performed using pmCherry-SEMI1-PAC as the template and PrimeSTAR Max DNA polymerase and the following primer sets containing overlapping sequences: 109–120Δ, #37 and #38; 273–279Δ, #39 and #40; and 645–655Δ, #41 and #42 (see below). Amplified fragments were used to transform NEB 5-alpha *E. coli* cells. The resulting plasmids (pmCherry-109–120Δ-PAC, pmCherry-273–279Δ-PAC, and pmCherry-645–655Δ-PAC) were linearized by digestion with SacI and KpnI before biolistic transformation. Protein expression was induced in cells by adding 75 ng/mL $CdCl_2$ during starvation.

#	Primer name	Sequence (5′→3′)
37	semi1_109–120Δ_F	GCTTGAAAATGATTTTATTAGCCATGAATTTTTTG
38	semi1_109–120Δ_R	AAATCATTTTCAAGCATTTACTATTCATTTAAATCCT
39	semi1_273–279Δ_F	TATTTTAAGATTTTTTAATTCTTTTTAAATGAGTTATGATAC
40	semi1_273–279Δ_R	AAAAATCTTAAAATATGATTTTCTTAAAACAAAACATTTTAAC
41	semi1_645–655Δ_F	AATTTTTGTGAACATCTCAGACATATGG
42	semi1_645–655Δ_R	ATGTTCACAAAAATTATTGATTGTTAGTCTTCAGG

DAPI staining

A suspension of cells was fixed by the addition of formaldehyde and Triton X-100 (final concentrations of 4% and of 0.5%, respectively). After careful mixing, cells were incubated for 30 min at room temperature and then centrifuged. The cell pellet was resuspended in 1/10 volume of 4% formaldehyde + 3.4% sucrose. A total of 80 μL of this mixture was spread onto a clean slide and air-dried. For chromosome staining, slides were incubated for 10 min in phosphate buffered saline (PBS) and mounted under a coverslip in Vectashield anti-fading agent (Vector Laboratories, Burlingame, CA, USA, H-1000) containing 50 μg/mL DAPI.

Immunocytology

For immunostaining of Rad51, slides prepared by the same method as for DAPI staining were incubated for 10 min in PBS containing 0.05% Triton X-100 and then in PBS. An anti-Rad51 antibody (1:100 dilution; mouse monoclonal, Lab Vision/NeoMarkers, Fermont, CA, USA, RRID: AB_144075) was then applied, and incubated under a coverslip overnight at 4°C. The slides were then rinsed with PBS for 10 min. FITC-labeled goat anti-mouse antibody (1:500 dilution; Merck Millipore, Burlington, MA, USA, RRID: AB_92634) was applied and incubated under a coverslip at room temperature for 1 h in the dark. Finally, the slides were incubated twice for 10 min in PBS and mounted under a coverslip in Vectashield anti-fading agent containing 50 μg/mL DAPI. For γH2AX and H3K56ac immunostaining, cells were fixed in methanol at −20°C for 1 h. After removal of methanol by centrifugation (3000 × g, 1 min), the cell pellet was postfixed in 1% paraformaldehyde dissolved in PBS at 4°C for 1 h. After the removal of paraformaldehyde by centrifugation, the pellet was resuspended in PBS and incubated for 1 h at room temperature with primary antibodies: anti-γH2AX (1:500 dilution; mouse monoclonal; BioLegend, San Diego, CA, USA, RRID: AB_315794) and anti-H3K56ac (1:500 dilution; rabbit polyclonal; Active Motif, Carlsbad, CA, USA, RRID: AB_2661786) antibodies. After washing with PBS, cells were incubated with FITC-labeled goat anti-mouse (1:500 dilution) and Rhodamine-labeled goat anti-rabbit (1:2000 dilution; Merck Millipore, RRID: AB_90296) secondary antibodies for 1 h at room temperature in the dark. After washing with PBS, cells were resuspended in Vectashield anti-fading agent containing 50 μg/mL DAPI, dropped onto a slide and mounted under a coverslip.

Fluorescence microscopy of living cells

Living cells in 10 mM Tris-HCl (pH 7.4) were incubated with Hoechst33342 (50 ng/mL; Invitrogen, Carlsbad, CA, USA, Cat. H3570) at 30°C for 30 min. After incubation, the cells were concentrated by centrifugation, resuspended in 3% polyethylene oxide to increase the viscosity of the medium, and 1 μL of the cell suspension was placed into a Commodore Compressor device (Yan et al., 2014) to immobilize the cells for microscopic inspection.

Western blotting

Cells were fixed with 10% (w/v) trichloroacetic acid (TCA) to prevent proteolysis and incubated on ice for 30 min. After removal of TCA by centrifugation at 9000 × g for 1 min, cell pellets were lysed in polyacrylamide gel electrophoresis (PAGE) sample buffer (6% SDS, 6% 2-mercaptoethanol, 5% glycerol 36% urea, and 360 mM Tris- HCl, pH6.8) and boiled at 98°C for 3 min; 10 μg total protein was loaded into each lane of Mini-PROTEAN TGX Precast Gel (4–15%; Bio-Rad, Hercules, CA, USA, Cat. 4561083),

separated by SDS-PAGE, and transferred onto a polyvinylidene fluoride membrane (Bio-Rad). Membranes were washed in PBS-T (0.05% Tween 20 in PBS), blocked in 5% dry skimmed milk in PBS-T for 30 min, and incubated for 1 h at room temperature with anti-flag (1:2000; mouse monoclonal; Sigma-Aldrich, RRID: AB_259529), anti-RFP (1:2000; mouse monoclonal; ChromoTek, Planegg-Martinsried, Germany, RRID: AB_2631395), or anti-tubulin α (1:10,000; mouse monoclonal; Lab Vision/NeoMarkers, RRID: AB_144075) antibody. After washing in PBS-T, membranes were incubated in PBS-T containing 5% dry skimmed milk and horseradish peroxidase-conjugated goat anti-mouse IgG antibody (1:5000; Bio-Rad, RRID: AB_808614) for 1 h at room temperature. Membranes were washed with PBS-T and developed using Clarity Western ECL (Bio-Rad, Cat. 1705060). Restore Western Blot Stripping Buffer (Thermo Fisher Scientific, Waltham, MA, USA, 21059) was used to reprobe membranes.

Co-immunoprecipitation and mass spectrometry

For co-immunoprecipitation, mCherry-Semi1-expressing cells were pretreated with 0.5 mM phenylmethylsulfonyl fluoride (PMSF; Cell Signaling Technology, Danvers, MA, USA, Cat. 8553S) for 30 min at 30°C (Iwamoto et al., 2017) and then collected by centrifugation at 700 × g for 3 min. The cells were resuspended at 1.5×10^7 cells/mL in homogenization buffer composed of 150 mM NaCl, 1% Triton X-100, 2 mM PMSF, and Complete Protease Inhibitor Cocktail (Sigma-Aldrich, P8215-1ML), and homogenized by gentle pipetting on ice for 30 min. The lysate obtained after clarification at 10,000 × g for 15 min was incubated with 25 μL RFP-Trap magnetic agarose beads (ChromoTek, Cat. rtma-20; pretreated with 5 mM Sulfo-NHS-Acetate [Thermo Fisher Scientific, Cat. 26777]) at 4°C for 1 h. After three washes with 150 mM NaCl, the beads bearing immunoprecipitated proteins were submitted to the Mass Spectrometry Facility of the Max F. Perutz Laboratories (Vienna, Austria). To identify significant interaction partners from the affinity purification data, MS data were analyzed using SAINTexpress (Teo et al., 2014). The average SAINT scores were calculated for two experimental samples for each bait analyzed and a p value of <0.05 was considered to indicate a biologically significant interaction.

EdU incorporation assay

WT CU428.2 or *semi1*Δ mating type VI cells were incubated overnight in 10 mM Tris-HCl (pH 7.4) containing 50 μM 5-ethynyl-2′-deoxyuridine (EdU, Thermo Fisher Scientific, C10337). The cells were then washed with fresh 10 mM Tris-HCl (pH 7.4) and mixed with B2086 or *semi1*Δ mating type IV cells. After 5 h and 10 h, 30 μL cell suspension was put onto poly-L-lysine-coated slides and air-dried. The slides were then soaked in fixation solution (50 mM glycine dissolved in ethanol, pH 2.0) for 20 min at -20°C and then washed in PBS for 10 min at room temperature. Click-iT EdU Alexa Fluor reaction cocktail (Thermo Fisher Scientific, C10337) was applied and incubated under a coverslip for 30 min at room temperature. The slides were washed twice for 10 min in PBS and mounted under a coverslip in Vectashield anti-fading agent containing 50 μg/mL DAPI.

Supplemental References

AKEMATSU, T., FINDLAY, A., FUKUDA, Y., PEARLMAN, R. E., LOIDL, J., ORIAS, E. & HAMILTON, E. P. 2018. Resistance to 6-Methylpurine is Conferred by Defective Adenine Phosphoribosyltransferase in Tetrahymena. *Genes (Basel),* 9**,** 179.

AKEMATSU, T., FUKUDA, Y., GARG, J., FILLINGHAM, J. S., PEARLMAN, R. E. & LOIDL, J. 2017. Post-meiotic DNA double-strand breaks occur in Tetrahymena, and require Topoisomerase II and Spo11. *Elife,* 6.

BOLDRIN, F., SANTOVITO, G., FORMIGARI, A., BISHARYAN, Y., CASSIDY-HANLEY, D., CLARK, T. G. & PICCINNI, E. 2008. MTT2, a copper-inducible metallothionein gene from Tetrahymena thermophila. *Comp Biochem Physiol C Toxicol Pharmacol,* 147**,** 232-40.

CASSIDY-HANLEY, D., BOWEN, J., LEE, J. H., COLE, E., VERPLANK, L. A., GAERTIG, J., GOROVSKY, M. A. & BRUNS, P. J. 1997. Germline and Somatic Transformation of Mating Tetrahymena thermophila by Particle Bombardment. *Genetics,* 146**,** 135-147.

HAYASHI, A. & MOCHIZUKI, K. 2015. Targeted Gene Disruption by Ectopic Induction of DNA Elimination in Tetrahymena. *Genetics,* 201**,** 55-64.

IWAMOTO, M., MORI, C., HIRAOKA, Y. & HARAGUCHI, T. 2014. Puromycin resistance gene as an effective selection marker for ciliate Tetrahymena. *Gene,* 534**,** 249-55.

IWAMOTO, M., MORI, C., KOJIDANI, T., BUNAI, F., HORI, T., FUKAGAWA, T., HIRAOKA, Y. & HARAGUCHI, T. 2009. Two distinct repeat sequences of Nup98 nucleoporins characterize dual nuclei in the binucleated ciliate tetrahymena. *Curr Biol,* 19**,** 843-7.

IWAMOTO, M., OSAKADA, H., MORI, C., FUKUDA, Y., NAGAO, K., OBUSE, C., HIRAOKA, Y. & HARAGUCHI, T. 2017. Compositionally distinct nuclear pore complexes of functionally distinct dimorphic nuclei in the ciliate Tetrahymena. *J Cell Sci,* 130**,** 1822-1834.

KATAOKA, K., SCHOEBERL, U. E. & MOCHIZUKI, K. 2010. Modules for C-terminal epitope tagging of Tetrahymena genes. *J Microbiol Methods,* 82**,** 342-6.

LEE, S. R. & COLLINS, K. 2007. Physical and functional coupling of RNA-dependent RNA polymerase and Dicer in the biogenesis of endogenous siRNAs. *Nat Struct Mol Biol,* 14**,** 604-10.

MOCHIZUKI, K. 2008. High efficiency transformation of Tetrahymena using a codon-optimized neomycin resistance gene. *Gene,* 425**,** 79-83.

MOCHIZUKI, K., NOVATCHKOVA, M. & LOIDL, J. 2008. DNA double-strand breaks, but not crossovers, are required for the reorganization of meiotic nuclei in Tetrahymena. *J Cell Sci,* 121**,** 2148-58.

SHANG, Y., SONG, X., BOWEN, J., CORSTANJE, R., GAO, Y., GAERTIG, J. & GOROVSKY, M. A. 2002. A robust inducible-repressible promoter greatly facilitates gene knockouts, conditional expression, and overexpression of homologous and heterologous genes in Tetrahymena thermophila. *Proc Natl Acad Sci U S A,* 99**,** 3734-9.

TEO, G., LIU, G., ZHANG, J., NESVIZHSKII, A. I., GINGRAS, A. C. & CHOI, H. 2014. SAINTexpress: improvements and additional features in Significance Analysis of INTeractome software. *J Proteomics,* 100**,** 37-43.

YAN, Y., JIANG, L., AUFDERHEIDE, K. J., WRIGHT, G. A., TEREKHOV, A., COSTA, L., QIN, K., MCCLEERY, W. T., FELLENSTEIN, J. J., USTIONE, A., ROBERTSON, J. B., JOHNSON, C. H., PISTON, D. W., HUTSON, M. S., WIKSWO, J. P., HOFMEISTER, W. & JANETOPOULOS, C. 2014. A microfluidic-enabled mechanical microcompressor for the immobilization of live single- and multi-cellular specimens. *Microsc Microanal,* 20**,** 141-51.
